# Supplementary material for: Cranial anatomy of Bolotridon frerensis, an enigmatic cynodont from the Middle Triassic of South Africa, and its phylogenetic significance
Source: PeerJ. 2021 Jun 16;9:e11542. doi: 10.7717/peerj.11542 (PMC8214396; doi:10.7717/peerj.11542)
Supplement: Supplemental Information 1 [file peerj-09-11542-s001.pdf]

APPENDIX 1

List of characters used in the phylogenetic analysis of Huttenlocker & Sidor (2020), largely modified from that of Abdala (2007) and Kammerer (2016) with the addition of various adapted and new characters (sources detailed below). We deleted character 64, since it was present twice (27 and 64) in their analysis. The updated character list now consists of 110 craniodental and postcranial characters scored for 26 therapsid taxa (now including 16 cynodonts with the inclusion of *Bolotridon*, coded based on BSPG 1934-VIII-7). Characters coded '?' are either unknown or not applicable.

The following characters K1-K62 are from Kammerer (2016). (Originally adapted from the character matrices of Botha et al., 2007 and Abdala, 2007)

1. [K1] Snout in relation to the temporal region: longer (0), subequal (1), shorter (2).

BSPG 1934-VIII-7= ?

2. [K2] Septomaxilla facial process: long (0), short (1).

BSPG 1934-VIII-7= 1

3. [K3] Contact between the nasal and lacrimal: absent (0), present (1).

BSPG 1934-VIII-7= 1

4. [K4] Incisive foramen: absent/open, fissure-like (0), present/enclosed as foramen (1).

BSPG 1934-VIII-7= ?

5. [K5] Contact between vomer and maxilla in the palate: absent (0), present (1), maxilla covers vomer (2).

BSPG 1934-VIII-7= ?

6. [K6] Osseous secondary palate: absent (0), maxillo-palatine extensions present, but do not contact medially (1), complete, without contribution of the palatine (2), complete, with contribution of the palatine (3).

BSPG 1934-VIII-7= ?

7. [K7] Ectopterygoid: contacts maxilla (0), does not contact maxilla (1), absent (2).

BSPG 1934-VIII-7= ?

8. [K8] Palatal teeth: on the pterygoid and palatine (0), on the transverse process of the pterygoid (1), on the pterygoid boss (2), absent (3).

BSPG 1934-VIII-7= 3

9. [K9] Interpterygoid vacuity in adults: present (0), highly reduced or absent (1). (Modified from Kammerer, 2016 to reflect highly reduced state in *Lumkuia* and early cynodonts.)

BSPG 1934-VIII-7= 1

10. [K10] Boss/crest anterior to interpterygoid vacuity: reduced or absent (0), well developed (1)

BSPG 1934-VIII-7= 0

11. [K11] Suborbital vacuity in palate: absent (0), present (1). (Same as Hopson and Barghusen, 1986, char. 10.1 and Huttenlocker, 2013, char. 40.)

BSPG 1934-VIII-7= 0

12. [K12] Frontal in orbital margin: included (0), excluded (1).

BSPG 1934-VIII-7= 1

13. [K13] Frontal–epipterygoid contact: absent (0), present (1).

BSPG 1934-VIII-7= ?

14. [K14] Parietal foramen: present (0), absent (1).

BSPG 1934-VIII-7= ?

15. [K15] Postfrontal: present (0), absent (1).

BSPG 1934-VIII-7= 1

16. [K16] Postorbital bar: complete narrow (0), incomplete (1), complete wide (2).

BSPG 1934-VIII-7= 0

17. [K17] Parietal region: at the same level as the skull profile (0), high (1).

BSPG 1934-VIII-7= ?

18. [K18] Temporal region transverse width: widest in the middle of the zygoma (0), or widest more posteriorly (1). (Character phrasing slightly modified from K18.)

BSPG 1934-VIII-7= ?

19. [K19] Zygomatic arch dorsoventral height: slender (0), moderately deep (1), very deep (2).

BSPG 1934-VIII-7= ?

20. [K20] Infraorbital process: absent (0), suborbital angulation between maxilla and jugal present (1), descendant process of the jugal present (2).

BSPG 1934-VIII-7= 0

21. [K21] Inferior margin of the jugal in the zygoma: poorly developed longitudinally (0), well developed longitudinally almost reaching the posterior end of the zygoma (1).

BSPG 1934-VIII-7= ?

22. [K22] Posterior extension of the squamosal dorsal to the squamosal sulcus: absent (0), incipient (1), well developed (2). (Similar to Hopson and Barghusen, 1986, character 37.3.)

BSPG 1934-VIII-7= ?

23. [K23] Occipital crests: non-confluent anteromedially (0), confluent (1).

BSPG 1934-VIII-7= ?

24. [K24] Posttemporal fossa large axis in relation to the diameter of the foramen magnum: of the same size or slightly smaller (0), notably smaller (1). (Similar to character 37.2 of Hopson and Barghusen, 1986.)

BSPG 1934-VIII-7= ?

25. [K25] Paroccipital process in the base of the posttemporal fossa: present (0), absent (1).

BSPG 1934-VIII-7= ?

26. [K26] Tuberculum spheno-occipital (‘basal tubera’ of some authors): present (0), absent or highly reduced (1). (Character statements modified from K26.)

BSPG 1934-VIII-7= ?

27. [K27] Pterygoid quadrate ramus: present (0), absent (1).

BSPG 1934-VIII-7= ?

28. [K28] Epipterygoid ascending process: rodlike (0), moderately expanded (1), greatly expanded (2).

BSPG 1934-VIII-7= ?

29. [K29] Lateral flange of the prootic: absent (0), present (1).

BSPG 1934-VIII-7= ?

30. [K30] Pterygo-paraoccipital foramen: absent (0), present (1).

BSPG 1934-VIII-7= ?

31. [K33] Quadrate notch in the squamosal: absent (0), present (1).

BSPG 1934-VIII-7= ?

32. [K34] Stapes: perforated (0), unperforated (1).

BSPG 1934-VIII-7= ?

33. [K35] Jugular foramen: faces posteriorly (0), ventrally (1).

BSPG 1934-VIII-7= ?

34. [K36] Mastoid and quadrate processes of the paroccipital process: undifferentiated (0), differentiated (1).

BSPG 1934-VIII-7= ?

35. [K37] Occipital condyle: single (0), double (1).

BSPG 1934-VIII-7= ?

36. [K38] Mandibular symphysis: unfused (0), fused (1).

BSPG 1934-VIII-7= 1

37. [K40] Angular region of dentary position relative to postorbital bar: anterior to the postorbital bar (0), at the same level or posterior (1).

BSPG 1934-VIII-7= 1

38. [K41] Longitudinal depression in the lateral side of the dentary: absent (0), present (1).

BSPG 1934-VIII-7= 0

39. [K42] Location of the coronoid process in the temporal fossa: lateral (0), in the middle (1).

BSPG 1934-VIII-7= ?

40. [K43] Mandibular fenestra open on external surface of the lower jaw between dentary and angular: absent (0), present (1).

BSPG 1934-VIII-7= ?

41. [K44] Reflected lamina of the angular: corrugated plate (0), smooth plate with slight depressions (1), hook-like laminae (2), thin projection (3).

BSPG 1934-VIII-7= ?

42. [K45] Masseteric fossa in the dentary: absent (0), fossa high on coronoid process (1), fossa extends to the angle of dentary (2).

BSPG 1934-VIII-7= 2

43. [K46] Position of the dentary/surangular dorsal contact: closer to postorbital bar (0), midway (1), closer to jaw joint (2).

BSPG 1934-VIII-7= ?

44. [K47] Surangular–squamosal contact: absent (0), present (1).

BSPG 1934-VIII-7= ?

45. [K48] Upper incisors: more than four (0), four (1).

BSPG 1934-VIII-7= 1

46. [K49] Lower incisors: four (0), three (1).

BSPG 1934-VIII-7= 1

47. [K50] Incisor cutting margins: serrated (0), smoothly ridged (1).

BSPG 1934-VIII-7= ?

48. [K51] Upper incisor–canine diastema: present (0), absent (1).

BSPG 1934-VIII-7= 0

49. [K52] Precanine maxillary teeth, absent (0), present (1).

BSPG 1934-VIII-7= 0

50. [K53] Lower canine: large (0), reduced (1).

BSPG 1934-VIII-7= 0

51. [K54] Canine serrations: present (0), absent (1).

BSPG 1934-VIII-7= 1

52. [K55] Upper postcanine row posterior extent: anterior to the orbit (0), below the orbit (1).

BSPG 1934-VIII-7= 1

53. [K56] Upper postcanine morphology: conical simple (0), sectorial (1), transversely wide (2), sectorial with lingual cingulum (3).

BSPG 1934-VIII-7= 1

54. [K57] Postcanine occlusion absence/presence: absent (0), present (1).

BSPG 1934-VIII-7= 0

55. [K58] Lingual cingulum in lower postcanines: absent (0), present (1).

BSPG 1934-VIII-7= 1

56. [K59] Posterior postcanines with strongly curved main cusp: absent (0), present (1).

BSPG 1934-VIII-7= 0

57. [K60] Marked diastema or step between maxillary canine and postcanines: absent (0), present (1).

BSPG 1934-VIII-7= 0

58. [K61] Dorsoventral maxillary depression posterior to canine root: absent (0), present (1).

BSPG 1934-VIII-7= 0

59. [K62] Dentary symphysis morphology: low, gently sloping, does not form distinct ‘chin’ (0), tall, steeply-sloped, forms distinct ‘chin’ (1).

BSPG 1934-VIII-7= 0

Characters A10-A87 are sampled from the character matrix of Abdala (2007). Redundant characters already adapted for characters 1-59 (above) are excluded below.

60. [A10] Contact between postorbital and squamosal: present (0), absent (1).

BSPG 1934-VIII-7= ?

61. [A21] Maxilla participation on anterior margin of subtemporal fenestra: excluded (0), included (1).

BSPG 1934-VIII-7= 0

62. [A27] Carotid artery foramina in basisphenoid: present (0), absent (1).

BSPG 1934-VIII-7= ?

63. [A28] Parasphenoid ala: long and borders fenestra ovalis (0), slightly reduced and excluded from fenestra ovalis (1). (Modified from Abdala (2007) char. 28; state 2 is omitted here.)

BSPG 1934-VIII-7= ?

64. [A31] Quadrate rami of epipterygoid: absent (0), present but do not contact quadrate (1), present and contact quadrate (2).

BSPG 1934-VIII-7= ?

65. [A33] Paroccipital process: does not contact quadrate (0), contacts quadrate (1), crista parotica contacts quadrate (2).

BSPG 1934-VIII-7= ?

66. [A37] Prootic and opisthotic: separated (0), fused to form petrosal (1).

BSPG 1934-VIII-7= ?

67. [A48] Lateral crest of dentary: absent (0), incipient (1), well developed (2), strongly projected (3).

BSPG 1934-VIII-7= 1

68. [A50] Base of coronoid process extension in lateral view: relatively narrow (0), moderately expanded anteroposteriorly (1), very expanded anteroposteriorly (2).

BSPG 1934-VIII-7= ?

69. [A59] Craniomandibular articulation: quadrate ⁄ articular (0), main quadrate ⁄ articular, secondary surangular ⁄ squamosal (1).

BSPG 1934-VIII-7= ?

70. [A66] Trigeminal exit: between prootic incisure and epipterygoid (0), via fenestra between epipterygoid and prootic (1), via two foramina (2).

BSPG 1934-VIII-7= ?

71. [A73] Latero-posterior exposure of squamosal on zygoma: without or with incipient depression (0), with deep squamosal sulcus (1).

BSPG 1934-VIII-7= ?

72. [A75] V-shape notch separating lambdoidal crest from zygoma: absent (0), incipient (1), deep (2).

BSPG 1934-VIII-7= ?

73. [A87] Axis of posterior part of maxillary tooth row: directed lateral to subtemporal fossa (0), directed toward centre of fossa (1), directed toward medial rim of fossa and curved (2), directed toward medial rim of fossa and parallel (3).

BSPG 1934-VIII-7= 0

Characters HK82-HK101 are postcranial features sampled from Hopson & Kitching (2001).

74. [HK82] Expanded costal plates on ribs: absent (0), present (1).

BSPG 1934-VIII-7= ?

75. [HK83] Lumbar costal plates with ridge overlapping preceding rib: absent (0), present (1).

BSPG 1934-VIII-7= ?

76. [HK84] Scapula infraspinous fossa with outturned anterior and posterior borders: absent (0), present (1).

BSPG 1934-VIII-7= ?

77. [HK85] Acromion process: absent (0), present (1).

BSPG 1934-VIII-7= ?

78. [HK88] Procoracoid in glenoid: present (0), barely present or absent (1).

BSPG 1934-VIII-7= ?

79. [HK90] Humerus ectepicondylar foramen: present (0), absent (1).

BSPG 1934-VIII-7= ?

80. [HK92] Manual digit III phalanx number: four (0), three (1).

BSPG 1934-VIII-7= ?

81. [HK93] Manual digit IV phalanx number: five (0), four (1), three (2).

BSPG 1934-VIII-7= ?

82. [HK94] Length of anterior process of ilium anterior to acetabulum (relative to diameter of acetabulum): less than 1.0 (0), 1.0-1.5 (1), greater than 1.5 (2).

BSPG 1934-VIII-7= ?

83. [HK95] Length of posterior process of ilium posterior to acetabulum: (relative to diameter of acetabulum): between 0.5 and 1.0 (0), greater than 1.0 (1), less than 0.5 (2).

BSPG 1934-VIII-7= ?

84. [HK96] Dorsal profile of ilium: strongly convex (0), flat to concave (1).

BSPG 1934-VIII-7= ?

85. [HK97] Total length of pubis relative to acetabulum diameter: greater than 1.5 (0), between 1.5 and 1.0 (1), less than 1.0 (2).

BSPG 1934-VIII-7= ?

Characters N1-N25 are additional cynodont and non-cynodont theriodont characters that were newly adapted by Huttenlocker & Sidor (2020). Some are modified from various sources indicated below (Hopson and Barghusen, 1986; Huttenlocker, 2013; Huttenlocker and Sidor, 2016; Benoit et al., 2017), while some were newly added by Huttenlocker & Sidor (2020).

86. [N1] Parabasisphenoid ventromedian keel: prominent, anteroposteriorly long (0), reduced to narrow process (1), absent (2).

BSPG 1934-VIII-7= ?

87. [N2] Interclavicle: elongated, strap-like element (0), short, shield-shaped (1).

BSPG 1934-VIII-7= ?

88. [N3] Ossified sternum: absent (0), present (1).

BSPG 1934-VIII-7= ?

89. [N4] Intercrista breadth shortest between maxillae (0), between palatine processes (1). (Applicable to taxa retaining a fissure-like choana and lacking a fully-formed secondary palate.)

BSPG 1934-VIII-7= ?

90. [N5] Vomer interchoana process narrow, parallel-sided (0), widens anteriorly (1). (Newly adapted therocephalian character from Huttenlocker, 2013.)

BSPG 1934-VIII-7= 0

91. [N6] Stapes dorsal process present (0), reduced or absent (1). (Newly adapted therocephalian character from Huttenlocker, 2013.)

BSPG 1934-VIII-7= ?

92. [N7] Iliac blade with anterior process and notch absent (0), present (1). (Newly adapted therocephalian character from Huttenlocker, 2013.)

BSPG 1934-VIII-7= ?

93. [N8] Femur with prominent trochanter minor and internal trochanter absent (0), present (1). (Newly adapted therocephalian character from Huttenlocker, 2013.)

BSPG 1934-VIII-7= ?

94. [N9] Incisor texture smooth (0), fluted or faceted (1).

BSPG 1934-VIII-7= 0

95. [N10] Distal carpals IV and V separate (0), fused (1).

BSPG 1934-VIII-7= ?

96. [N11] Orbitosphenoid ossification (modified from Benoit et al., 2017): present and well ossified (0), absent/unossified (1), weakly ossified, cancellous (2).

BSPG 1934-VIII-7= ?

97. [N12] Orbitosphenoid anteroposterior length (modified from Benoit et al., 2017): elongated (0), reduced/absent (1).

BSPG 1934-VIII-7= ?

98. [N13] Articulation of the orbitosphenoids to the skull roofing elements (modified from

Benoit et al., 2017): sutural connection present (0), loose, not articulated (1).

BSPG 1934-VIII-7= ?

99. [N14] Interorbital septum (‘presphenoid’) keel bony contributions: exists as a separate median ossification (0), absent/unossified (1), continuous with the paired orbitosphenoids as a single Y-shaped element (2). (The baurioid therocephalians *Tetracynodon* and *Mupashi* are not coded in present matrix; Huttenlocker and Sidor, 2016).

BSPG 1934-VIII-7= ?

100. [N15] Diastema between lower canine and postcanines short to absent (0), present and long, up to the length of two to three tooth positions (1).

BSPG 1934-VIII-7= 0

101. [N16] Postcanine row medially inset absent (0), present (1).

BSPG 1934-VIII-7= 0

102. [N17] Splenial obscured completely by dentary in lateral view: no, visible along ventral portion of mandible (0), yes, splenial is obscured from lateral view (1).

BSPG 1934-VIII-7= 1

103. [N18] Anterodorsal process of prootic with extensive epipterygoid contact: contact limited (0), present, contact extensive (1). (Newly adapted from Hopson and Barghusen, 1986.)

BSPG 1934-VIII-7= ?

104. [N19] Paroccipital process anteroposterior depth: anteroposteriorly narrow (0), anteroposteriorly expanded (1). (Newly adapted from Hopson and Barghusen, 1986.)

BSPG 1934-VIII-7= ?

105. [N20] Unossified gap present between prootic anterodorsal process and parietal/interparietal: present (0), mostly closed by sutural union of these elements (1).

BSPG 1934-VIII-7= ?

106. [N21] Zygomatic arch greatest width at: middle (0), or at back of arch, forming a triangular dorsal skull profile (1). (Newly adapted therocephalian character from Huttenlocker, 2013.)

BSPG 1934-VIII-7= ?

107. [N22] Stapedial foramen: present (0), absent (1). (Newly adapted therocephalian character from Huttenlocker, 2013.)

BSPG 1934-VIII-7= ?

108. [N23] Squamosal medial process contacting prootic: absent (0), contact present and

enclosing the pterygo-paroccipital foramen (1). (Newly adapted therocephalian character from Huttenlocker, 2013.)

BSPG 1934-VIII-7= ?

109. [N24] Vomer anterior vault: present (0), absent (1). (Newly adapted therocephalian character from Huttenlocker, 2013.)

BSPG 1934-VIII-7= ?

110. [N25] Atlas-axis fusion: Atlas centrum separate (0), fused or partially fused to axis to form a “dens-like” structure (1).

BSPG 1934-VIII-7= ?

APPENDIX 2

PAUP* output.

```
paup> contree;

Strict consensus of 24 trees:

/--------------------------------------------------------------------------------------------------------------------------------- Cyonosaurus
|
+--------------------------------------------------------------------------------------------------------------------------------- Aelurognathus
|
|                                                                                          /-------------------------------------- Lycosuchus
|                                                                                          |
|                                                                                          |            /------------------------- Glanosuchus
|            /-----------------------------------------------------------------------------+            |
|            |                                                                             |            |            /------------ Olivierosuchus
|            |                                                                             |            |            |
|            |                                                                             \------------+            +------------ Moschorhinus
|            |                                                                                          |            |
|            |                                                                                          |            +------------ Hofmeyria
|            |                                                                                          \------------+
|            |                                                                                                       +------------ Theriognathus
|            |                                                                                                       |
|            |                                                                                                       +------------ Regisaurus
|            |                                                                                                       |
|            |                                                                                                       \------------ Bauria
|            |
|            |                         /------------------------------------------------------------------------------------------ Procynosuchus
|            |                         |
\------------+                         |                                                                             /------------ Vetusodon
             |                         |                         /---------------------------------------------------+
             |                         |                         |                                                   \------------ Cynosaurus
             |                         |                         |
             |                         |                         |            /--------------------------------------------------- Thrinaxodon
             |            /------------+            /------------+            |
             |            |            |            |            |            |                                      /------------ Nanictosaurus
             |            |            |            |            |            +--------------------------------------+
             |            |            |            |            |            |                                      \------------ Platycraniellus
             |            |            |            |            \------------+
             |            |            |            |                         |                                      /------------ Cynognathus
             |            |            |            |                         |                         /------------+
             |            |            \------------+                         |                         |            \------------ Diademodon
             |            |                         |                         |            /------------+
             \------------+                         |                         \------------+            \------------------------- Lumkuia
                          |                         |                                      |
                          |                         |                                      \-------------------------------------- Bolotridon
                          |                         |
                          |                         |                                                                /------------ Galesaurus
                          |                         \----------------------------------------------------------------+
                          |                                                                                          \------------ Progalesaurus
                          |
                          |                                                                /-------------------------------------- Dvinia
                          |                                                                |
                          \----------------------------------------------------------------+            /------------------------- Charassognathus
                                                                                           |            |
                                                                                           \------------+            /------------ Abdalodon
                                                                                                        \------------+
                                                                                                                     \------------ Nshimbodon
```

```
;

Bootstrap method with heuristic search:
  Number of bootstrap replicates = 1000
  Starting seed = generated automatically
  Number of characters resampled in each replicate = 110
  Optimality criterion = parsimony
    Character-status summary:
      Of 110 total characters:
        All characters are of type 'unord'
        All characters have equal weight
        1 character is constant (proportion = 0.00909091)
        2 variable characters are parsimony-uninformative
        Number of parsimony-informative characters = 107
    Gaps are treated as "missing"
    Multistate taxa interpreted as polymorphism
  Starting tree(s) obtained via stepwise addition
    Addition sequence: simple (reference taxon = Cyonosaurus)
    Number of trees held at each step = 1
  Branch-swapping algorithm: tree-bisection-reconnection (TBR) with reconnection limit = 8
    Steepest descent option not in effect
  Initial 'Maxtrees' setting = 2600 (will be auto-increased by 100)
  Branches collapsed (creating polytomies) if maximum branch length is zero
  'MulTrees' option in effect
  No topological constraints in effect
  Trees are unrooted

  1000 bootstrap replicates completed
  Time used = 00:32:47 (CPU time = 00:32:34.1)
```

Bootstrap 50% majority-rule consensus tree

```
/--------------------------------------------------------------------------------------------------------------------------------- Cyonosaurus(1)
|
+--------------------------------------------------------------------------------------------------------------------------------- Aelurognathus(2)
|
|                                                                       /--------------------------------------------------------- Lycosuchus(3)
|                                                                       |
|                                                                       |             /------------------------------------------- Glanosuchus(4)
|             /----------------------------85---------------------------+             |
|             |                                                         |             |             /----------------------------- Olivierosuchus(5)
|             |                                                         \-----73------+             |
|             |                                                                       |             +----------------------------- Moschorhinus(6)
|             |                                                                       |             |
|             |                                                                       |             +----------------------------- Hofmeyria(7)
|             |                                                                       \-----76------+
|             |                                                                                     +----------------------------- Theriognathus(8)
|             |                                                                                     |
|             |                                                                                     |              /-------------- Regisaurus(9)
|             |                                                                                     \------52------+
|             |                                                                                                    \-------------- Bauria(10)
|             |
|             |                            /-------------------------------------------------------------------------------------- Procynosuchus(11)
\-----97------+                            |
              |                            |             /------------------------------------------------------------------------ Vetusodon(13)
              |                            |             |
              |              /-----68------+             +------------------------------------------------------------------------ Cynosaurus(14)
              |              |             |             |
              |              |             |             |                                                         /-------------- Galesaurus(15)
              |              |             |             +----------------------------79---------------------------+
              |              |             |             |                                                         \-------------- Progalesaurus(16)
              |              |             \-----99------+
              |              |                           |              /--------------------------------------------------------- Thrinaxodon(17)
              |              |                           |              |
              |              |                           |              |                                          /-------------- Nanictosaurus(18)
              |              |                           |              +---------------------55-------------------+
              |              |                           |              |                                          \-------------- Platycraniellus(19)
              \-----100------+                           \------57------+
                             |                                          |                                          /-------------- Cynognathus(20)
                             |                                          |                           /-----100------+
                             |                                          |                           |              \-------------- Diademodon(21)
                             |                                          |             /-----93------+
                             |                                          \-----66------+             \----------------------------- Lumkuia(22)
                             |                                                        |
                             |                                                        \------------------------------------------- Bolotridon(26)
                             |
                             |                                                        /------------------------------------------- Dvinia(12)
                             |                                                        |
                             \----------------------------51--------------------------+             /----------------------------- Charassognathus(23)
                                                                                      |             |
                                                                                      \-----98------+              /-------------- Abdalodon(24)
                                                                                                    \------91------+
                                                                                                                   \-------------- Nshimbodon(25)
```

Bipartitions found in one or more trees and frequency of occurrence (bootstrap support values):

```
         1         2     2
12345678901234567890123456          Freq        %
-----------------------------------------------------
..........****************       1000.00  100.00%
...................**.....        998.23   99.82%
............**********...*        994.13   99.41%
......................***.        975.40   97.54%
..************************        970.90   97.09%
...................***....        934.35   93.43%
.......................**.        912.95   91.30%
..********................        850.73   85.07%
..............**..........        794.19   79.42%
....******................        755.72   75.57%
...*******................        727.68   72.77%
..........*.**********...*        683.31   68.33%
...................***...*        662.52   66.25%
................******...*        566.41   56.64%
.................**.......        549.79   54.98%
........**................        517.92   51.79%
...........*..........***.        513.41   51.34%
....**....................        391.12   39.11%
......**..................        341.40   34.14%
................***.......        330.12   33.01%
....***...................        285.16   28.52%
..........**..............        282.72   28.27%
..............********...*        261.22   26.12%
......***.................        250.90   25.09%
............**............        243.83   24.38%
............**..******...*        223.96   22.40%
.......**.................        222.51   22.25%
............*.********...*        211.48   21.15%
.............*********...*        197.37   19.74%
....****..................        191.60   19.16%
....*****.................        188.89   18.89%
............*...******...*        188.48   18.85%
.......***................        187.68   18.77%
..............*****.......        184.05   18.41%
.....***..................        181.92   18.19%
................***......*        177.92   17.79%
..........************...*        175.93   17.59%
...***********************        146.18   14.62%
............**************        146.14   14.61%
....*.****................        138.56   13.86%
....**********************        137.79   13.78%
.................*****...*        133.52   13.35%
..............*****......*        130.89   13.09%
............**.....***....        127.83   12.78%
....*...**................        123.31   12.33%
....*....*................        119.76   11.98%
............**.....***...*        116.41   11.64%
............*......***....        113.97   11.40%
.....**...................        113.58   11.36%
..**......................        108.68   10.87%
............*......***...*         91.96    9.20%
..............**..*.......         89.25    8.93%
..........*.**************         86.11    8.61%
.................**......*         78.69    7.87%
..........**..........***.         74.16    7.42%
................**........         73.91    7.39%
................*.*.......         64.02    6.40%
..**....**................         62.51    6.25%
.....****.................         55.39    5.54%
....**....****************         52.97    5.30%

541 groups at (relative) frequency less than 5% not shown
```

```
paup> pscores / CI RI;

Lengths and fit measures of trees in memory:
  Character-status summary:
    Of 110 total characters:
      All characters are of type 'unord'
      All characters have equal weight
      1 character is constant (proportion = 0.00909091)
      2 variable characters are parsimony-uninformative
      Number of parsimony-informative characters = 107
  Gaps are treated as "missing"
  Multistate taxa interpreted as polymorphism

  Sum of min. possible lengths = 153
  Sum of max. possible lengths = 691

Tree #      1
Length    256
CI      0.598
RI      0.809
```

[illegible]
RI   0.809

APPENDIX 3

MrBayes output.

```
Logging screen output to file "CharassBayes.log"
Setting Rates to Gamma
Successfully set likelihood model parameters
WARNING: There is one character incompatible with the specified
         coding bias. This character will be excluded.
Setting autoclose to yes
Setting number of generations to 1000000
Setting print frequency to 1000
Setting sample frequency to 100
Setting number of chains to 4
Setting chain output file names to "CharassBayes.run<i>.<p/t>"
Successfully set chain parameters
Running Markov chain
MCMC stamp = 4635027396
Seed = 1676304429
Swapseed = 1614094084
```

```
Clade credibility values:

/---------------------------------------------------------- Cyonosaurus (1)
|
|---------------------------------------------------------- Aelurognathus (2)
|
|                                  /----------------------- Lycosuchus (3)
|                                  |
|                                  |     /----------------- Glanosuchus (4)
|     /-------------90-------------+     |
|     |                            |     |          /------ Olivierosuchus (5)
|     |                            |     |    /--82-+
|     |                            \--91-+    |     \------ Moschorhinus (6)
|     |                                  |    |
|     |                                  |    |------------ Hofmeyria (7)
+     |                                  \-92-+
|     |                                       |------------ Theriognathus (8)
|     |                                       |
|     |                                       |     /------ Regisaurus (9)
|     |                                       \--57-+
|     |                                             \------ Bauria (10)
|     |
|     |                /----------------------------------- Procynosuchus (11)
|     |                |
|     |                |                            /------ Vetusodon (13)
|     |                |     /----------71----------+
|     |                |     |                      \------ Cynosaurus (14)
\--69-+          /--99-+     |
      |          |     |     |                      /------ Galesaurus (15)
      |          |     |     |----------81----------+
      |          |     |     |                      \------ Progalesaurus (16)
      |          |     |     |
      |          |     \-100-+     /----------------------- Thrinaxodon (17)
      |          |           |     |
      |          |           |     |                /------ Nanictosaurus (18)
      |          |           |     |-------68-------+
      |          |           |     |                \------ Platycraniellus (19)
      |     /-60-+           |     |
      |     |    |           \--99-+                /------ Cynognathus (20)
      |     |    |                 |          /-100-+
      |     |    |                 |          |     \------ Diademodon (21)
      |     |    |                 |     /-94-+
      |     |    |                 |     |    \------------ Lumkuia (22)
      |     |    |                 \--97-+
      \-100-+    |                       \----------------- Bolotridon (26)
            |    |
            |    \----------------------------------------- Dvinia (12)
            |
            |                                 /------------ Charassognathus (23)
            |                                 |
            \----------------93---------------+     /------ Abdalodon (24)
                                              \--94-+
                                                    \------ Nshimbodon (25)
```

```
Phylogram (based on average branch lengths):


|
|- Aelurognathus (2)
|
|          /- Lycosuchus (3)
|          |
|          |   / Glanosuchus (4)
|  /-------+   |
|  |       |   |    /- Olivierosuchus (5)
|  |       |   |  /-+
|  |       \---+  | \ Moschorhinus (6)
|  |           |  |
|  |           |  |- Hofmeyria (7)
+  |           \--+
|  |              |- Theriognathus (8)
|  |              |
|  |              |/- Regisaurus (9)
|  |              \+
|  |               \------- Bauria (10)
|  |
|  |                            /- Procynosuchus (11)
|  |                            |
|  |                            |          /- Vetusodon (13)
|  |                            |         /+
|  |                            |         |\- Cynosaurus (14)
\--+                        /---+         |
   |                        |   |         |/ Galesaurus (15)
   |                        |   |         |+
   |                        |   |         |\- Progalesaurus (16)
   |                        |   |         |
   |                        |   \---------+ / Thrinaxodon (17)
   |                        |             | |
   |                        |             | |/ Nanictosaurus (18)
   |                        |             | |+
   |                        |             | |\-- Platycraniellus (19)
   |                     /--+             | |
   |                     |  |             \-+              /-- Cynognathus (20)
   |                     |  |               |     /--------+
   |                     |  |               |     |        \---- Diademodon (21)
   |                     |  |               | /---+
   |                     |  |               | |   \--- Lumkuia (22)
   |                     |  |               \-+
   \---------------------+  |                 \- Bolotridon (26)
                         |  |
                         |  \-- Dvinia (12)
                         |
                         |   /- Charassognathus (23)
                         |   |
                         \---+ / Abdalodon (24)
                             \-+
                               \ Nshimbodon (25)

|----------| 0.200 expected changes per site

Calculating tree probabilities...

Credible sets of trees (10440 trees sampled):
   50 % credible set contains 2939 trees
   90 % credible set contains 8940 trees
   95 % credible set contains 9690 trees
   99 % credible set contains 10290 trees
```

REFERENCES CITED IN SUPPLEMENTARY INFORMATION

Abdala F. 2007. Redescription of *Platycraniellus elegans* (Therapsida, Cynodontia) from the Lower Triassic of South Africa, and the cladistic relationships of eutheriodonts. *Palaeontology* 50(3):591–618 DOI 10.1111/j.1475-4983.2007.00646.x.

Abdala F, Gaetano LC, Smith RM, Rubidge BS. 2019. A new large cynodont from the Late Permian (Lopingian) of the South African Karoo Basin and its phylogenetic significance. *Zoological Journal of the Linnean Society* 186(4):983–1005 DOI: 10.1093/zoolinnean/zlz004.

Benoit J, Jasinoski S, Fernandez V, Abdala F. 2017. The mystery of a missing bone: revealing the orbitosphenoid in basal Epicynodontia (Cynodontia, Therapsida) through computed tomography. *The Science of Nature* 104(7–8):66 DOI: 10.1007/s00114-017-1487-z.

Botha, J., F. Abdala, and R. Smith. 2007. The oldest cynodont: new clues on the origin and earlydiversification of the Cynodontia. Zoological Journal of the Linnean Society 149(3):477–492.

Hopson JA, Barghusen H.R.. 1986. An analysis of therapsid relationships. In: Hotton N, MacLean PD, Roth JJ, Roth EC, eds. *The Ecology and Biology of Mammal-like Reptiles.* Washington and London: Smithsonian Institution Press, 83–106.

Hopson JA, Kitching JW. 2001. A probainognathian cynodont from South Africa and the phylogeny of nonmammalian cynodonts. *Bulletin of the Museum of Comparative Zoology* 156(1):5–35.

Huttenlocker AK. 2013. Paleobiology of therocephalian synapsids (Amniota) and the effects of the end-Permian extinction on size, growth and bone microstructure. Doctoral dissertation, Ph. D. dissertation, University of Washington, Seattle.

Huttenlocker AK, Abdala F. 2015. Revision of the first therocephalian, *Theriognathus* Owen (Therapsida: Whaitsiidae), and implications for cranial ontogeny and allometry in nonmammaliaform eutheriodonts. *Journal of Paleontology* 89(4):645–664.

Huttenlocker AK, Sidor CS. 2016. The first karenitid (Therapsida, Therocephalia) from the upper Permian of Gondwana and the biogeography of Permo-Triassic therocephalians. *Journal of Vertebrate Paleontology* 36(4):e1111897.

Huttenlocker AK, Sidor CA. 2020. A basal nonmammaliaform cynodont from the Permian of Zambia and the origins of mammalian endocranial and postcranial anatomy. *Journal of Vertebrate Paleontology* e1827413 DOI 10.1080/ 02724634.2020.1827413.

Kammerer CF. 2016. A new taxon of cynodont from the *Tropidostoma* Assemblage Zone (upper Permian) of South Africa, and the early evolution of Cynodontia. *Papers in Palaeontology* 2(3):387–397 DOI 10.5061/dryad.52064.
